# Depressive mood mediates the influence of social support on health-related quality of life in elderly, multimorbid patients

**DOI:** 10.1186/1471-2296-15-62

**Published:** 2014-04-08

**Authors:** Felix S Wicke, Corina Güthlin, Karola Mergenthal, Jochen Gensichen, Christin Löffler, Horst Bickel, Wolfgang Maier, Steffi G Riedel-Heller, Siegfried Weyerer, Birgitt Wiese, Hans-Helmut König, Gerhard Schön, Heike Hansen, Hendrik van den Bussche, Martin Scherer, Anne Dahlhaus

**Affiliations:** 1 Institute of General Practice, Goethe University Frankfurt, Frankfurt am Main, Germany; 2 Department of General Practice, Jena University Hospital, Jena, Germany; 3 Institute of General Practice, Rostock University Medical Center, Rostock, Germany; 4 Department of Psychiatry, Technical University of Munich, Munich, Germany; 5 Department of Psychiatry and Psychotherapy, University of Bonn, Bonn, Germany; 6 Institute of Social Medicine, Occupational Health and Public Health, University of Leipzig, Leipzig, Germany; 7 Central Institute of Mental Health, Medical Faculty Mannheim/Heidelberg University, Mannheim, Germany; 8 Institute for Biometry, Hannover Medical School, Hannover, Germany; 9 Department of Medical Sociology and Health Economics, Hamburg Center for Health Economics, University Medical Center Hamburg-Eppendorf, Hamburg, Germany; 10 Department of Medical Biometry and Epidemiology, Hamburg-Eppendorf University Medical Center, Hamburg, Germany; 11 Department of Primary Medical Care, Hamburg-Eppendorf University Medical Center, Hamburg, Germany

**Keywords:** Multimorbidity, Chronic medical conditions, Coping, Primary care, Family practice, Social support, Health-related quality of life, Depression, Elderly patients

## Abstract

**Background:**

It is not well established how psychosocial factors like social support and depression affect health-related quality of life in multimorbid and elderly patients. We investigated whether depressive mood mediates the influence of social support on health-related quality of life.

**Methods:**

Cross-sectional data of 3,189 multimorbid patients from the baseline assessment of the German MultiCare cohort study were used. Mediation was tested using the approach described by Baron and Kenny based on multiple linear regression, and controlling for socioeconomic variables and burden of multimorbidity.

**Results:**

Mediation analyses confirmed that depressive mood mediates the influence of social support on health-related quality of life (Sobel’s p < 0.001). Multiple linear regression showed that the influence of depressive mood (β = −0.341, p < 0.01) on health-related quality of life is greater than the influence of multimorbidity (β = −0.234, p < 0.01).

**Conclusion:**

Social support influences health-related quality of life, but this association is strongly mediated by depressive mood. Depression should be taken into consideration in research on multimorbidity, and clinicians should be aware of its importance when caring for multimorbid patients.

**Trial registration:**

ISRCTN89818205

## Background

Health-related quality of life is a measure of subjective health that complements disease-specific outcomes in multimorbid patients, because good quality of life is of value in itself and it is an independent predictor of mortality [1,2]. All relevant factors that might affect health-related quality of life in multimorbid patients need to be looked at in order to gain a more detailed biopsychosocial understanding [3] of multimorbidity. These include biological factors such as the extent of multimorbidity, as well as psychosocial factors such as social support and depression. The present study aims to clarify the relationship between quality of life, depression and social support in multimorbid patients.

Multimorbidity is commonly defined as the co-occurrence of two or more diseases and medical conditions within one person [4]. As the number of simultaneous chronic diseases increases with age, multimorbidity is common in elderly patients [5,6]. In a recent study the prevalence of multimorbidity in primary care practices was 65% in elderly persons [5]. Previous research has attempted to increase our understanding of multimorbidity by identifying patterns of disease combinations [7-9]. Diseases tend to co-occur when they share common risk factors or pathophysiological pathways [10], or, given the high prevalence of many diseases in the elderly, by coincidence. Based on factor-analysis, Schäfer et al. [9] identified three multimorbidity patterns: cardiovascular/metabolic disorders (CMD), anxiety/depression/somatoform disorders and pain (ADS/P) and neuropsychiatric disorders (NPS).

Multimorbidity has several adverse consequences for patients, e.g. polypharmacy and decreases in functional abilities. Furthermore, it is known that the burden of multimorbidity consistently leads to impaired health-related quality of life in primary care patients [11,12].

Social support by relatives, friends or professionals can promote adaptation to and coping with chronic illness and multimorbidity [13,14]. Social support is a broad concept that is commonly divided into instrumental and emotional support, as well as into actually provided, received, and perceived social support [15]. Perceived social support can be defined as ‘the perception or experience that one is loved and cared for by others, esteemed and valued, and part of a social network of mutual assistance and obligations’ (Wills cited in [15]). Social support is positively correlated with health-related quality of life [16,17] and was identified by Fortin et al. [11] to be one of the most important factors predicting health-related quality of life in multimorbid patients. Therefore, understanding how social support and quality of life are related, can inform primary care interventions addressing multimorbid patients.

Social support is negatively correlated with depression [18,19]. Impaired social support and feelings of loneliness are considered to be risk factors for depression in the elderly [20,21]. As social support is associated both with health-related quality of life and with depression, the question arises whether and how these factors interact.

Psychological distress [22] and depression [23,24] are known to affect health-related quality of life. This association is especially important in multimorbid patients because duration and severity of depression have a higher negative impact on health-related quality of life than physical chronic conditions [25]. Additionally, depression is a common [26] and often chronic [27] comorbidity in elderly patients. And, in primary care patients, the probability of suffering from depression grows with increasing physical morbidity [28].

In previous research, depression was shown to be a mediating variable in the relationship between social support and health-related quality of life in patients with HIV/AIDS [18,29]. As an explanation, Bekele et al. have proposed that either a perceived lack of social support increases perceived threats of stressful events, or a high level of perceived social support decreases perceived threats of stressful events [29]. This, in turn, leads to either an increase or decrease in depressive symptoms, and influences health-related quality of life accordingly. As social support has a positive and depression a detrimental effect on health-related quality of life in multimorbid patients, a similar relationship to that observed in patients with HIV/AIDS may exist.

**Figure 1:**
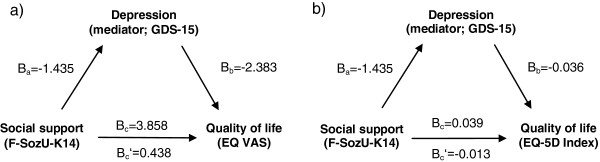
**Analytic relation between study variables.** Legend: Analytical relation between social support (F-SozU-K14), depressive mood (GDS-15) and health-related quality of life (outcome variable EQ VAS **(a)** and EQ-5D Index **(b)**). Values are unstandardized regression coefficients.

To clarify the direct and indirect effects of social support and depressive mood on health-related quality of life in multimorbid patients, we investigated the hypothesis that depressive mood mediates the influence of social support on health-related quality of life in these patients. Additionally, we investigated whether the hypothesis holds true for the three different multimorbidity patterns. The analytic relation is depicted in Figure 1.

## Methods

Cross-sectional data come from the baseline assessment of the German MultiCare study, a longitudinal, prospective observational cohort study of multimorbid elderly patients [30]. 3,189 patients were recruited from 158 general practices in eight study centres across Germany. The study protocol was approved by the Ethics Committee of the Medical Association of Hamburg.

### Participants and sampling

Included patients were between 65 and 85 years of age, had visited their general practitioner (GP) at least once within the previous three-month period and had at least three chronic medical conditions from a list of 29 common diseases. Patients were randomly selected based on medical records from primary care practices but were excluded if they were unable to participate in interviews (deaf, blind or unable to speak German), if they were not regular patients of the respective practice, if they were living in nursing homes, if they were not able to give informed consent (e.g. demented patients), or if they had an acute illness which was expected to result in death within three months. The complete list of diseases and further details on the study design can be found elsewhere [30,31]. Data were obtained from GPs’ medical records and from standardized comprehensive interviews with patients.

### Measures

#### Depressive mood

We used the Geriatric Depression Scale, which was developed for assessment of depression in elderly persons [32]. It avoids assessment of physical symptoms, which in elderly and comorbid patients cannot clearly be attributed to depression [33]. Validation studies of the Geriatric Depression Scale in hospitals and nursing-home residents showed good results [34]. In primary care populations, it appears to be preferable to use the short version of the Geriatric Depression Scale (GDS-15) instead of the long version [35], and hence the short version was used in this study. The German version of the GDS-15, used here, showed good psychometric properties [36]. The scale comprises 15 items that can be answered either with yes or no, with a threshold score of ≥6 out of 15 making major depression likely [36]. As others have done before, we used the GDS-15 as a continuous scale in our mediator analysis to assess for depressive mood [24], based on the assumption that a higher score on the GDS-15 reflects greater depressive mood than a lower score, regardless of the threshold.

#### Social support

To assess perceived social support, the short form of the Social Support Questionnaire was used (F-SozU-K14). The F-SozU-K14 is commonly used in Germany (e.g. [37]) and good evidence for its validity exists [38]. A continuous summary score is calculated from its 14 items, with higher values indicating more perceived social support. The F-SozU-K14 assesses perceived emotional support, perceived practical support and perceived social integration. However, for the short form of the Social Support Questionnaire, no differentiation to these subscales is recommended by the authors [39], which is why the summary score was used.

#### Health-related quality of life

Health-related quality of life was measured using the EuroQol-5D-3L (EQ-5D) instrument [40]. Patients were asked to self-rate their current health state on a visual analogue scale (EQ VAS) from 0 to 100. Additionally, the EQ-5D assesses five dimensions of the current health state of patients: mobility, self-care, usual activities, pain/discomfort, and anxiety/depression. Each dimension is assessed on three levels: no problems, some problems, or severe problems. Thereby a total of 243 possible health states results, from which a single continuous index score can be obtained (EQ-5D Index; 1 represents perfect health and 0 represents death). This is carried out using health-state valuation data from valuation studies in general populations. We used the European valuation data based on Greiner et al. [41] reported in Szende et al. [42]. In our analyses we used both the EQ VAS and the EQ-5D Index variables to assess the outcome of health-related quality of life.

The EQ-5D is among the briefest health index measures and due to its ease of application has high completion rates in elderly populations, but it has been criticized for being less sensitive to change than the SF-36 [43]. In a multimorbid patient sample, however, its lower sensitivity is probably less relevant, because patients are relatively sick and inter-individual differences more pronounced [34]. We therefore consider the EQ-5D to be a reliable and valid instrument for a multimorbid patient sample.

#### Control variables

Socioeconomic control variables used were: age, gender, educational level, income and living-situation. Educational level was divided into three categories, based on the CASMIN-classification [44]: 1) inadequately completed general education, general elementary education or basic vocational qualification; 2) intermediate qualification or general maturity certificate; 3) lower or higher tertiary education. Income was reported as household-size adjusted net income per month. Participants were classified as either ‘living with a partner or relative’, or as ‘living alone’. ‘Living alone’ included assisted living or living in retirement homes.

As a control variable for the disease burden of multimorbidity, a weighted disease count was included in the model. There is no consensus on how to measure multimorbidity and many different measures exist [45]. Because a multimorbidity measure incorporating severity of disease was described to be associated with psychological distress, while a simple disease count was not [22], it seemed appropriate to account for disease severity. In this study, patients’ diagnoses and severity of diseases were assessed in interviews with GPs. The weighted disease count was then calculated by summing up the severity ratings (‘marginal’ = 0, ‘low’ = 1, ‘medium’ = 2, ‘severe’ = 3 and ‘very severe’ = 4) given by the physician. Pearson’s correlation of the weighted and unweighted disease count was r = 0.774 (p < 0.01).

To test the mediation hypothesis for different multimorbidity patterns, patients were assigned to the three patterns described above (CMD, ADS/P and NPS) if they had at least three diseases belonging to one of these groups, as described by Schäfer et al. [9].

### Missing values

Missing data were imputed using the hot deck method from donors, and identified on the basis of the nearest Gower distance. 2,720 patients (85.3%) had no missing values and were eligible as donors. Eight participants with missing data needed for the calculation of the EQ-5D Index variable were excluded from further analysis. A more detailed description of the imputation process can be found elsewhere [31].

### Statistical analyses

A correlation matrix was calculated using Pearson’s coefficients for continuous variables or Spearman’s coefficients for nominal and ordinal variables. To assess both direct and indirect effects of social support on health-related quality of life, we tested the mediation hypothesis as described by Baron and Kenny [46]. This approach is a measurement-of-mediation design. It is used to statistically measure the mediator variable’s effect, in contrast to experimental approaches like the experimental-chain-design, where the mediator variable is directly manipulated [47]. We used multiple linear regression to calculate unstandardized and standardized coefficients of variables as well as adjusted R-squares (R^2^). We confirmed assumptions for regression analyses by checking for linearity between variables based on plotting and curve-fitting procedures, by excluding multicollinearity based on variance inflation factors, and by assessing normal distribution of residuals graphically. Control variables were used in all regression calculations. Testing mediation requires three regression models, in which the following conditions must be fulfilled: first, the predictor variable (social support) must significantly influence the mediator variable (depressive mood); second, the predictor variable must significantly influence the outcome variable (health-related quality of life); and third, the predictor variable’s influence on the outcome variable must be greatly reduced or become non-significant when the mediator variable is included in the model. Mediation analyses were done separately for the two outcome variables EQ VAS and EQ-5D Index. To test significance of the indirect path via the mediator variable, we used Sobel’s test applying a utility provided by Preacher and Leonardelli [48]. To investigate whether the mediation hypothesis holds true in different multimorbidity patterns, we also conducted the mediation analyses separately for all patients exclusively assigned to the CMD pattern and for all patients exclusively assigned to the ADS/P pattern. Based on the assumption that differences would be more pronounced in patients that were assigned to one pattern alone, all patients assigned to multiple patterns were excluded. The NPS pattern was not accounted for, as only four patients were exclusively assigned to it. All resulting values were said to be significant at a level of p < 0.05. Analyses were done using SPSS version 19.0.

## Results

### Sample characteristics

The total sample at baseline consisted of 3,189 patients. The mean age was 74.4 years and 59.3% of patients were female (see Table 1). 62.3% of patients had a low educational status. The mean number of chronic conditions was 7.0. The three most common diagnoses were hypertension, disorders of lipid metabolism and chronic low back pain. A more detailed description of age, gender, and socio-economic characteristics of the study cohort can be found elsewhere [31].

**Table 1:** Characteristics of the study population and multimorbidity patterns

	**All (n = 3,189)**	**CMD (n = 937)**	**ADS/P (n = 748)**
Age: mean (SD)	74.4 (5.2) years	74.3 (5.2) years	73.8 (5.2) years
Gender: N (%) female	1,891 (59.3)	362 (38.6)	607 (81.1)
Living situation: N (%)			
With partner or family member	2,000 (62.7)	635 (67.8)	424 (56.7)
Alone, assisted living or retirement home	1,189 (37.3)	302 (32.2)	324 (43.3)
Education: N (%)			
Low	1,986 (62.3)	591 (63.1)	439 (58.7)
Medium	856 (26.8)	227 (24.2)	244 (32.6)
High	347 (10.9)	119 (12.7)	65 (8.7)
Income: mean (SD)	1,412 (704) Euros	1,433 (838) Euros	1,404 (596) Euros
Number of chronic conditions (SD)	7.0 (2.5)	6.2 (1.5)	6.2 (1.6)
Prevalence of most common conditions			
Hypertension	77.9%	90.0%	54.4%
Disorders of lipid metabolism	58.5%	68.5%	36.6%
Chronic low back pain	49.5%	24.3%	62.7%
Depression			
Prevalence	17.7%	6.1%	25.4%
Mean GDS-15 (SD)	2.57 (2.60)	2.35 (2.36)	2.45 (2.60)
Perceived Social Support			
F-SozU-K14: mean (SD)	4.1 (0.69)	4.11 (0.66)	4.14 (0.68)
Health-related quality of life			
EQ VAS: mean (SD)	62.4 (18.2)	64 (18)	64 (18)
EQ-5D Index: mean (SD)	0.70 (0.23)	0.75 (0.22)	0.70 (0.22)

SD: Standard deviation.

CMD: cardiovascular/metabolic disorders pattern.

ADS/P: anxiety/depression/somatoform disorders and pain pattern.

In the analysis of multimorbidity patterns, 937 patients were exclusively assigned to the CMD pattern and 748 patients to the ADS/P pattern. Further characteristics on the subsamples are displayed in Table 1.

Based on the GDS-15, 401 patients were likely to have depression. Of those, 144 (36%) had previously been diagnosed with depression, according to GP records. Patients that were likely to have depression consistently reported more problems in all five dimensions of health-related quality of life than patients unlikely to have depression.

### Correlations

We examined correlations among variables for magnitude and plausibility with regard to our hypothesis (Table 2). Social support correlated negatively with burden of multimorbidity and depressive mood, and positively with both measures of health-related quality of life (EQ VAS and EQ-5D Index). Health-related quality of life, as measured either by the EQ VAS or the EQ-5D Index, correlated negatively with the weighted disease count and with depressive mood.

**Table 2:** Correlations and descriptive statistics of study variables

	**EQ VAS**	**EQ-5D Index**	**GDS-15**	**F-SozU-K14**	**Weighted disease count**	**Age**	**Gender**	**Educational level**	**Household income**	**Living with partner**	**Mean**	**Standard deviation**
EQ VAS	-										62.4	18.2
EQ-5D Index	.530^**^	-									0.703	0.23
GDS-15	-.406^**^	-.446^**^	-								2.57	2.60
F-SozU-K14	.178^**^	.140^**^	-.407^**^	-							4.01	0.69
Weighted disease count	-.315^**^	-.295^**^	.197^**^	-.076^**^	-						11.26	5.14
Age	-.112^**^	-.108^**^	.125^**^	-.121^**^	.162^**^	-					74.4	5.19
Gender (female = 1)^†^	-.060^**^	-.164^**^	.092^**^	.005	-.042^*^	.054^**^	-				-	-
Educational level^†^	.108^**^	.103^**^	-.068^**^	.016	-.117^**^	-.054^**^	-.111^**^	-			-	-
Household income	.127^**^	.086^**^	-.139^**^	.072^**^	-.076^**^	-.007	-.123^**^	.258^**^	-		1,412	706
Living with partner^†^	.062^**^	.070^**^	-.117^**^	.128^**^	-.028	-.191^**^	-.314^**^	.066^**^	.046^**^	-	-	-

**Correlation is significant at p < 0.01 (two-sided).

*Correlation is significant at p < 0.05 (two-sided).

^†^Spearman’s correlation coefficient. All others are Pearson’s coefficients.

### Mediator analyses

The predictor variable (social support) significantly influenced the proposed mediator (depressive mood) in a linear regression model; this first model is the same for both outcome variables. The unstandardized regression coefficient Ba was −1.435 (p < 0.001, standard error SE = 0.061). The coefficients used for testing mediation are also shown in Figure 1.

### Mediator analysis with EQ VAS

Our first analysis used EQ VAS as outcome variable. The predictor variable social support significantly influenced the outcome variable (step 1 in Table 3); the unstandardized coefficient Bc was 3.858 (p < 0.001, SE = 0.446). When adding the proposed mediator (depressive mood) to the linear regression model (step 2 in Table 3), the social support coefficient decreased to Bc’ = 0.438 (SE = 0.457) and lost statistical significance (p = 0.338), suggesting perfect mediation according to Baron and Kenny. Depressive mood significantly influenced the outcome variable with a coefficient Bb of −2.383 (p < 0.001, SE = 0.123). The indirect pathway was significant at p < 0.001, based on Sobel’s test.

**Table 3:** Effects of social support and depression on health-related quality of life estimated using linear regression

	**EQ VAS (N = 3,189)**				**EQ-5D Index (N = 3,181**^**†**^**)**			
	**Step 1**		**Step 2**		**Step 1**		**Step 2**	
**Predictors**	**B [95%-CI]**	**β**	**B [95%-CI]**	**β**	**B [95%-CI]**	**β**	**B [95%-CI]**	**β**
Social support	3.858** [2.983;4.733]	0.146**	0.438 [−0.458;1.334]	0.017	0.039** [0.028;0.050]	0.116**	−0.013* [−0.024;-0.002]	−0.038*
Depressive mood	-	-	−2.383** [−2.632;-2.143]	−0.341**	-	-	−0.036** [−0.039;-0.033]	−0.409**
Control variables								
Multimorbidity	−1.022** [−1.139;-0.904]	−0.288**	−0.830** [−0.942;-0.718]	−0.234**	−0.013** [−0.014;-0.011]	−0.281**	−0.010** [−0.011;-0.008]	−0.216**
Age	−0.149* [−0.267;-0.032]	−0.043*	−0.096 [−0.207;0.015]	−0.027	−0.002* [−0.003;0.000]	−0.042*	−0.001 [−0.002;0.000]	−0.024
Gender	−1.797* [−3.078;-0.516]	−0.049*	−0.846 [−2.061;0.369]	−0.023	−0.077** [−0.093;-0.061]	−0.165**	−0.063** [−0.078;-0.048]	−0.134**
Educational level	1.218* [0.317;2.120]	0.046*	1.424** [0.572;2.277]	0.054**	0.013* [−0.002;0.025]	0.040*	0.016** [0.006;0.027]	0.049**
Income	0.002** [0.001;0.003]	0.077**	0.001** [0.000;0.002]	0.044**	0.000 [0.000;0.000]	0.026	0.000 [0.000;0.000]	−0.013
Living with partner	−0.170 [−1.491;1.151]	−0.005	−0.520 [−1.769;0.729]	−0.014	−0.014 [0.030;0.003]	−0.028	−0.019* [−0.035;-0.004]	−0.040*
R^2^ (adjusted)	0.137**		0.228**		0.132**		0.264**	

**significant at p < 0.01.

*significant at p < 0.05.

^†^EQ-5D data was missing for eight participants and these data were not imputed.

B: unstandardized regression coefficient (95%-CI: 95%-confidence interval).

β: standardized regression coefficient.

### Mediator analysis with EQ-5D Index

The same analysis was done using the EQ-5D Index as outcome variable. Again, the predictor variable significantly influenced health-related quality of life (step 1 in Table 3). After adding depressive mood to the model, the unstandardized coefficient decreased from Bc = 0.039 (p < 0.001, SE = 0.006) to Bc’ = −0.013 (95%-CI: −0.024 to −0.002, SE = 0.006) and remained significant at p = 0.023 (step 2 in Table 3). Depressive mood still exerted significant influence on the EQ-5D Index variable with a coefficient Bb = −0.036 (p < 0.001, SE = 0.002). The indirect path was significant at p < 0.001, according to Sobel’s test.

Assessment of overall strength of our regression model showed that the addition of the mediator variable depressive mood to the model greatly increased R^2^ on both occasions: when using EQ VAS as outcome variable, R^2^ increased from 0.137 to 0.228 (Table 3) and when using EQ-5D Index, R^2^ increased from 0.132 to 0.264.

### Further variables

The weighted disease count as a measure of multimorbidity and level of education were the only other variables with a significant influence on health-related quality of life in both models after inclusion of depressive mood. Comparison of standardized regression coefficients showed health-related quality of life to be more strongly affected by depressive mood than by the weighted disease count (Table 3). The influence of level of education was marginal.

### Multimorbidity patterns

When conducting the mediation analysis in the multimorbidity patterns of CMD and ADS/P, results differed only marginally between the two groups and from the overall analysis. As no meaningful difference between patients in the various patterns was identified, results are not shown.

## Discussion

### Key findings

Our results show that depressive mood mediates the association between social support and health-related quality of life in multimorbid, elderly patients. This finding holds true in the overall sample and in the two groups of distinct multimorbidity patterns (CMD and ADSP). The fact that multimorbidity patterns did not differ significantly in our analysis suggests that no disease-specific mechanisms are at work, at least with regard to social support and health-related quality of life. In view of this, it may make more sense to consider general disease susceptibility, related to psychosocial factors such as coping style, as a possible explanation of the results [49]. In our analyses, all main criteria for mediation were fulfilled, including significance of the indirect path. Interestingly, the social support coefficient changed from positive to negative in the EQ-5D Index analysis, which may be the result of a suppressor effect. As it decreased markedly after accounting for depressive mood, and confidence intervals were close to zero, we interpreted this result to be in agreement with the mediation hypothesis.

In both analyses, the large increase in R^2^-values after accounting for depressive mood suggests that although the total effect of social support is mediated by depressive mood, the total effect of depressive mood cannot be explained through social support alone. Therefore additional unaccounted variables are of importance when predicting depressive mood in multimorbid patients.

In addition, our data showed that depressive mood affects health-related quality of life more than the overall disease burden of multimorbidity (as measured by the weighted disease count), which agrees with previous research [25]. For comparison with previous research it should be borne in mind that we have considered depression in late life to be a continuous concept. We therefore used a continuous score and did not dichotomize the GDS-15.

For a theoretical explanation of our results, we draw on cognitive appraisal theory, which is commonly applied in research on adaptation to chronic diseases [13,50]. According to this theory, a person’s encounter with stress leads to a primary appraisal of stressors, in this case multiple illnesses, and to a secondary appraisal of coping resources. An adverse primary appraisal of threat and harm resulting from multiple chronic diseases compounded by a perceived lack of social support in the secondary appraisal process could result in depressive mood. Conversely, the perception of good social support could balance a harmful primary appraisal. The level of depressive mood then influences health-related quality of life either positively or negatively. Cognitive appraisal takes part in coping with chronic diseases and knowledge of the processes involved can aid GPs when supporting patients [50].

To our knowledge, depression as a mediator between social support and health-related quality of life has previously only been studied in patients with HIV/AIDS: in the study by Jia et al. [18] the effect of social support on physical and mental health-related quality of life was completely mediated by depression. In contrast, in the study by Bekele et al. [29] mediation was not complete and a significant, yet small, direct effect of social support on health-related quality of life remained after accounting for depression. Sociodemographic and medical differences between the samples of HIV/AIDS patients and our sample limit direct comparison, but we believe that the similar findings in all samples support the hypothesis.

### Strengths and limitations

Major strengths of our study are its large sample size and its coverage of many different diseases. We consider our sample of 3,189 patients with a multitude of common diseases to be highly representative of elderly, multimorbid patients in primary care settings and would contend that our results are more suited to be generalized than studies limited to a single chronic disease. This is of great advantage especially for primary care practice.

Our study is limited by its cross-sectional design (longitudinal results will, however, be available from the MultiCare study in the future). Consequently, one criterion for mediation mentioned by Baron and Kenny [46] cannot be checked: namely that the mediator is not caused by the outcome variable. We cannot exclude this possibility as there is no way of knowing the sequence of events in our sample. Here, experimental designs to test mediation could provide stronger evidence of causal relations among variables [47]. We used a unique multimorbidity score, as described in detail in the methods section, which we consider to be superior to a simple disease count for this study. However, it limits comparison to other studies on multimorbidity. Given the multitude of multimorbidity scores [45], this is a common limitation of multimorbidity research.

### Implications for research

Future research needs to clarify and integrate further variables in a model of social support and health-related quality of life. Several other variables have been shown to act as mediators between social support and health-related quality of life: sense of coherence [51], self-esteem and control beliefs [52]. As shown by Schwarzer et al. [53], self-efficacy can act as an intermediate variable between social support and depression. Furthermore, personality traits such as neuroticism may affect all relevant variables: social support, depressive mood and self-ratings of quality of life [54,55]. Altogether this points to the complexity of what are often reciprocal relations. Ideally this should lead to integrated models that include all relevant variables and are tested in longitudinal rather than cross-sectional studies. The next step would be the design of clinical studies modifying one or more of the implicated variables in the attempt to improve quality of life of multimorbid patients in primary care.

### Implications for family practice

Our data add to existing evidence showing the importance of depression as comorbidity in patients with physical illness [23]. The relative importance of depressive mood compared to social support in our study’s results suggests that, regarding health-related quality of life, interventions directed at depressive mood are probably more effective than interventions to improve social support. As Löffler et al. showed, coping with multimorbidity is an active process by patients, often requiring the utilisation of all their resources including social support [14]. Reduced energy and decreased activity as hallmark symptoms of depression potentially limit the coping process with multimorbidity and therefore need to be addressed in primary care practice. Effective treatment can be limited by the failure to recognize depressive mood and clinical depression in practice. This can be very problematic in multimorbid patients, because symptoms of depression are easily misattributed to somatic illness. Even if diagnosed correctly, overall evidence on optimal management of depression in multimorbid patients is scarce [56] and multimorbidity is only poorly reflected in clinical guidelines [57]. Notable exceptions exist: randomised-controlled trials have shown that improvements in depressive symptoms [58-60] and in social activity [61] are achievable in primary practice settings. A stepped care approach in primary care was also shown to be effective to prevent late life depression [62]. Bogner et al. [58] and Katon et al. [59] showed that in patients with depression and a chronic physical condition, outcomes of both can be improved by integrated and collaborative care, where physicians receive guideline-based recommendations for treatment. Katon et al. also showed improvements in quality of life. In the study by Gensichen et al. [60] outcomes of depression were improved by structured telephone interviews to monitor depression symptoms and support for adherence to medication; quality of life, however, did not significantly improve. Although none of these studies addressed social support, Sommers et al. [61] showed increased social activity when involving social workers in collaborative care. These trials suggest that a promising way to improve outcomes in multimorbid patients with depression are integrated care strategies specifically addressing physical and mental conditions as well as social concerns.

## Conclusion

Social support influences health-related quality of life, but this association is strongly mediated by depressive mood. This finding can be explained by cognitive appraisal theory in the sense that social support either protects against or predisposes a person to depressive mood when faced with the threat of multiple illnesses, and this, in turn, affects health-related quality of life. Further research needs to integrate multiple psychosocial factors in order to explain health-related quality of life in multimorbid patients. Integrated care models that specifically address somatic, mental and social dimensions are promising in improving outcomes in multimorbid patients and interventional studies assessing all three dimensions are needed. In family practice, GPs should take into account social support (e.g. by family, friends and support groups) as a potential resource and depressive mood as impediment when caring for multimorbid patients.

## Competing interests

The authors declare that they have no competing interests.

## Authors' contributions

HvdB, KW, HH und IS designed the study. HvdB and MS are principal investigators of the study. KM, JG, CL, HB, WM, SGR-H, SW, BW, H-HK, GS, HH, HvdB, MS and AD participated in study design and implementation. FSW, CG and AD developed the hypothesis and planned statistical analysis. FSW conducted statistical analysis, prepared the first draft of the manuscript and was in charge of manuscript development and finalization. AD and CG were involved in preparation of the manuscript. All authors read and approved the final manuscript.

## Pre-publication history

The pre-publication history for this paper can be accessed here:

http://www.biomedcentral.com/1471-2296/15/62/prepub

## References

[B1] Drageset J Eide GE Nygaard HA Bondevik M Nortvedt MW Natvig GK The impact of social support and sense of coherence on health-related quality of life among nursing home residents–a questionnaire survey in Bergen, Norway Int J Nurs Stud 2009 46 65 75 18721922 10.1016/j.ijnurstu.2008.07.005

[B2] Haring R Feng Y Moock J Volzke H Dorr M Nauck M Wallaschofski H Kohlmann T Self-perceived quality of life predicts mortality risk better than a multi-biomarker panel, but the combination of both does best BMC Med Res Methodol 2011 11 103 10.1186/1471-2288-11-103 21749697 PMC3152941

[B3] Engel GL The need for a new medical model: a challenge for biomedicine Science 1977 196 129 136 10.1126/science.847460 847460

[B4] van den Akker M Buntinx F Knottnerus JA Comorbidity or multimorbidity: what's in a name? A review of literature Eur J Gen Pract 1996 2 65 70 10.3109/13814789609162146

[B5] Barnett K Mercer SW Norbury M Watt G Wyke S Guthrie B Epidemiology of multimorbidity and implications for health care, research, and medical education: a cross-sectional study Lancet 2012 380 37 43 10.1016/S0140-6736(12)60240-2 22579043

[B6] Rizza A Kaplan V Senn O Rosemann T Bhend H Tandjung R Age- and gender-related prevalence of multimorbidity in primary care: the Swiss FIRE project BMC Fam Pract 2012 13 113 10.1186/1471-2296-13-113 23181753 PMC3557138

[B7] García-Olmos L Salvador CH Alberquilla Á Lora D Carmona M García-Sagredo P Pascual M Muñoz A Monteagudo JL García-López F Comorbidity patterns in patients with chronic diseases in general practice PLoS ONE 2012 7 e32141 10.1371/journal.pone.0032141 22359665 PMC3281110

[B8] Kirchberger I Meisinger C Heier M Zimmermann A Thorand B Autenrieth CS Peters A Ladwig K Döring A Patterns of multimorbidity in the aged population. Results from the KORA-Age Study PLoS ONE 2012 7 e30556 10.1371/journal.pone.0030556 22291986 PMC3264590

[B9] Schäfer I von Leitner EC Schön G Koller D Hansen H Kolonko T Kaduszkiewicz H Wegscheider K Glaeske G van den Bussche H Multimorbidity patterns in the elderly: a new approach of disease clustering identifies complex interrelations between chronic conditions PLoS ONE 2010 5 e15941 10.1371/journal.pone.0015941 21209965 PMC3012106

[B10] Stolk R Rosmalen JM Postma D Boer R Navis G Slaets JJ Ormel J Wolffenbuttel BR Universal risk factors for multifactorial diseases Eur J Epidemiol 2008 23 67 74 10.1007/s10654-007-9204-4 18075776

[B11] Fortin M Bravo G Hudon C Lapointe L Almirall J Dubois M Vanasse A Relationship between multimorbidity and health-related quality of life of patients in primary care Qual Life Res 2006 15 83 91 10.1007/s11136-005-8661-z 16411033

[B12] Fortin M Lapointe L Hudon C Vanasse A Ntetu A Maltais D Multimorbidity and quality of life in primary care: a systematic review Health Qual Life Outcomes 2004 2 51 10.1186/1477-7525-2-51 15380021 PMC526383

[B13] Stanton AL Revenson TA Tennen H Health psychology: psychological adjustment to chronic disease Annu Rev Psychol 2007 58 565 592 10.1146/annurev.psych.58.110405.085615 16930096

[B14] Loffler C Kaduszkiewicz H Stolzenbach C Streich W Fuchs A van den Bussche H Stolper F Altiner A Coping with multimorbidity in old age - a qualitative study BMC Fam Pract 2012 13 45 10.1186/1471-2296-13-45 22639848 PMC3403868

[B15] Taylor SE Friedman HS Social Support: A Review The Oxford handbook of health psychology 2011 New York: Oxford University Press 189 214

[B16] Sherbourne CD Meredith LS Rogers W Ware JE Social support and stressful life events: age differences in their effects on health-related quality of life among the chronically ill Qual Life Res 1992 1 235 246 10.1007/BF00435632 1299454

[B17] Zhou ES Penedo FJ Lewis JE Rasheed M Traeger L Lechner S Soloway M Kava BR Antoni MH Perceived stress mediates the effects of social support on health-related quality of life among men treated for localized prostate cancer J Psychosom Res 2010 69 587 590 10.1016/j.jpsychores.2010.04.019 21109047 PMC2994072

[B18] Jia H Uphold CR Wu S Reid K Findley K Duncan PW Health-related quality of life among men with HIV infection: effects of social support, coping, and depression AIDS Patient Care ST 2004 18 594 603 10.1089/apc.2004.18.594 15630787

[B19] Lin N Ye X Ensel WM Social support and depressed mood: a structural analysis J Health Soc Behav 1999 40 344 359 10.2307/2676330 10643160

[B20] Blazer DG2 Hybels CF Origins of depression in later life Psychol Med 2005 35 1241 1252 10.1017/S0033291705004411 16168147

[B21] Theeke LA Goins RT Moore J Campbell H Loneliness, depression, social support, and quality of life in older chronically ill Appalachians J Psychol 2012 146 155 171 10.1080/00223980.2011.609571 22303618

[B22] Fortin M Bravo G Hudon C Lapointe L Dubois M Almirall J Psychological distress and multimorbidity in primary care Ann Fam Med 2006 4 417 422 10.1370/afm.528 17003141 PMC1578652

[B23] Moussavi S Chatterji S Verdes E Tandon A Patel V Ustun B Depression, chronic diseases, and decrements in health: results from the World Health Surveys Lancet 2007 370 851 858 10.1016/S0140-6736(07)61415-9 17826170

[B24] Wiesmann U Niehörster G Hannich H Subjective health in old age from a salutogenic perspective Br J Health Psychol 2009 14 767 787 10.1348/135910709X413124 19245743

[B25] Noël PH Williams JW Unützer J Worchel J Lee S Cornell J Katon W Harpole LH Hunkeler E Depression and comorbid illness in elderly primary care patients: impact on multiple domains of health status and well-being Ann Fam Med 2004 2 555 562 10.1370/afm.143 15576541 PMC1466751

[B26] Spangenberg L Forkmann T Brahler E Glaesmer H The association of depression and multimorbidity in the elderly: implications for the assessment of depression Psychogeriatrics 2011 11 227 234 10.1111/j.1479-8301.2011.00375.x 22151242

[B27] Licht-Strunk E van Marwijk HWJ Hoekstra T Twisk JWR de Haan M Beekman ATF Outcome of depression in later life in primary care: longitudinal cohort study with three years' follow-up BMJ 2009 338 a3079 10.1136/bmj.a3079 19188214 PMC2635593

[B28] Gunn JM Ayton DR Densley K Pallant JF Chondros P Herrman HE Dowrick CF The association between chronic illness, multimorbidity and depressive symptoms in an Australian primary care cohort Soc Psychiatry Psychiatr Epidemiol 2012 47 175 184 10.1007/s00127-010-0330-z 21184214

[B29] Bekele T Rourke SB Tucker R Greene S Sobota M Koornstra J Monette L Rueda S Bacon J Watson J Hwang SW Dunn J Guenter D The Positive Spaces Healthy Places Team Direct and indirect effects of perceived social support on health-related quality of life in persons living with HIV/AIDS AIDS Care 2012 25 1 10 22774876 10.1080/09540121.2012.701716

[B30] Schäfer I Hansen H Schon G Maier W Hofels S Altiner A Fuchs A Gerlach F Petersen J Gensichen J Schulz S Riedel-Heller S Luppa M Weyerer S Werle J Bickel H Barth K Konig H Rudolph A Wiese B Prokein J Bullinger M von dem Knesebeck O Eisele M Kaduszkiewicz H Wegscheider K van den Bussche H The German MultiCare-study: Patterns of multimorbidity in primary health care - protocol of a prospective cohort study BMC Health Serv Res 2009 9 145 10.1186/1472-6963-9-145 19671164 PMC3224741

[B31] Schäfer I Hansen H Schon G Hofels S Altiner A Dahlhaus A Gensichen J Riedel-Heller S Weyerer S Blank W Konig HH von dem Knesebeck O Wegscheider K Scherer M van den Bussche H Wiese B The influence of age, gender and socio-economic status on multimorbidity patterns in primary care. First results from the MultiCare Cohort study BMC Health Serv Res 2012 12 89 10.1186/1472-6963-12-89 22471952 PMC3348059

[B32] Yesavage JA Brink TL Rose TL Lum O Huang V Adey M O von L Development and validation of a geriatric depression screening scale: a preliminary report J Psychiat Res 1982 17 37 49 10.1016/0022-3956(82)90033-4 7183759

[B33] Creed F Dickens C Steptoe A Depression in the medically il Depression and physical illness 2007 Cambridge: Cambridge University Press 3 18

[B34] McDowell I Measuring health: A guide to rating scales and questionnaires 2006 3 Oxford: Oxford Univ. Press

[B35] Mitchell AJ Bird V Rizzo M Meader N Diagnostic validity and added value of the geriatric depression scale for depression in primary care: a meta-analysis of GDS30 and GDS15 J Affect Disorders 2010 125 10 17 10.1016/j.jad.2009.08.019 19800132

[B36] Gauggel S Birkner B Validität und Reliabilität einer deutschen Version der Geriatrischen Depressionsskala (GDS) Z Klin Psychol Psych 1999 28 18 27 10.1026//0084-5345.28.1.18

[B37] Wiczinski E Döring A John J Lengerke T Obesity and health-related quality of life: does social support moderate existing associations? Br J Health Psychol 2009 14 717 734 10.1348/135910708X401867 19187576

[B38] Knoll N Kienle R Fragebogenverfahren zur Messung verschiedener Komponenten sozialer Unterstützung: ein Überblick Z Med Psychol 2007 16 57 71

[B39] Fydrich T Sommer G Brähler E F-SozU - Handbuch http://www.unifr.ch/ztd/HTS/inftest/WEB-Informationssystem/de/4de001/4ca9c6dbe5964b329f6fc7fb5cd864b7/hb.htm

[B40] Rabin R Oemar M Oppe M EQ-5D-3L User Guide. Basic information on how to use the EQ-5D-3L instrument [http://www.euroqol.org/about-eq-5d/publications/user-guide.html]

[B41] Greiner W Weijnen T Nieuwenhuizen M Oppe S Badia X Busschbach J Buxton M Dolan P Kind P Krabbe P Ohinmaa A Parkin D Roset M Sintonen H Tsuchiya A Charro F A single European currency for EQ-5D health states Eur J Health Econ 2003 4 222 231 10.1007/s10198-003-0182-5 15609189

[B42] Szende A Oppe M Devlin NJ EQ-5D value sets: Inventory, comparative review, and user guide 2007 Dordrecht: Springer

[B43] Brazier JE Walters SJ Nicholl JP Kohler B Using the SF-36 and Euroqol on an elderly population Qual Life Res 1996 5 195 204 10.1007/BF00434741 8998488

[B44] Brauns H Steinmann S Educational reform in France, West-Germany and the United Kingdom: updating the CASMIN Educational Classification ZUMA-Nachrichten 1999 44 7 44

[B45] Huntley A Johnson R Purdy S Valderas J Salisbury C Measures of multimorbidity and morbidity burden for use in primary care and community settings: a systematic review and guide Ann Fam Med 2012 10 134 141 10.1370/afm.1363 22412005 PMC3315139

[B46] Baron RM Kenny DA The moderator-mediator variable distinction in social psychological research: conceptual, strategic, and statistical considerations J Pers Soc Psychol 1986 51 1173 1182 3806354 10.1037//0022-3514.51.6.1173

[B47] Wu A Zumbo B Understanding and using mediators and moderators Soc Indic Res 2008 87 367 392 10.1007/s11205-007-9143-1

[B48] Preacher KJ Leonardelli GJ Calculation for the Sobel test: An interactive calculation tool for mediation tests http://quantpsy.org/sobel/sobel.htm

[B49] van den Akker M Vos R Knottnerus JA In an exploratory prospective study on multimorbidity general and disease-related susceptibility could be distinguished J Clin Epidemiol 2006 59 934 939 10.1016/j.jclinepi.2006.02.009 16895816

[B50] Lazarus R Cohen F Stone GC, Adler NE, Cohen F Coping with the Stresses of Illness Health psychology: a handbook. Theories, applications, and challenges of a psychological approach to the health care system 1979 1 San Francisco: Jossey-Bass 217 254

[B51] Vogel I Miksch A Goetz K Ose D Szecsenyi J Freund T The impact of perceived social support and sense of coherence on health-related quality of life in multimorbid primary care patients Chronic Illn 2012 10.1177/1742395312445935 22517927

[B52] Warner L Schuz B Wurm S Ziegelmann J Tesch-Romer C Giving and taking–differential effects of providing, receiving and anticipating emotional support on quality of life in adults with multiple illnesses J Health Psychol 2010 15 660 670 10.1177/1359105310368186 20603289

[B53] Schwarzer R Knoll N Functional roles of social support within the stress and coping process: a theoretical and empirical overview Int J Psychol 2007 42 243 252 10.1080/00207590701396641

[B54] Kendler KS Kuhn J Prescott CA The interrelationship of neuroticism, sex, and stressful life events in the prediction of episodes of major depression Am J Psychiatry 2004 161 631 636 10.1176/appi.ajp.161.4.631 15056508

[B55] Swickert R Owens T The interaction between neuroticism and gender influences the perceived availability of social support Pers Indiv Differ 2010 48 385 390 10.1016/j.paid.2009.10.033

[B56] Smith SM Soubhi H Fortin M Hudon C O'Dowd T Managing patients with multimorbidity: systematic review of interventions in primary care and community settings BMJ 2012 345 e5205 10.1136/bmj.e5205 22945950 PMC3432635

[B57] Tinetti ME Bogardus ST Jr Agostini JV Potential pitfalls of disease-specific guidelines for patients with multiple conditions N Engl J Med 2004 351 2870 2874 10.1056/NEJMsb042458 15625341

[B58] Bogner HR Morales KH de Vries HF Cappola AR Integrated management of Type 2 Diabetes Mellitus and depression treatment to improve medication adherence: a randomized controlled trial Ann Fam Med 2012 10 15 22 10.1370/afm.1344 22230826 PMC3262455

[B59] Katon WJ Lin EH Von Korff M Ciechanowski P Ludman EJ Young B Peterson D Rutter CM McGregor M McCulloch D Collaborative care for patients with depression and chronic illnesses N Engl J Med 2010 363 2611 2620 10.1056/NEJMoa1003955 21190455 PMC3312811

[B60] Gensichen J von Korff M Peitz M Muth C Beyer M Güthlin C Torge M Petersen JJ Rosemann T König J Gerlach FM Case management for depression by health care assistants in small primary care practices: a cluster randomized trial Ann Intern Med 2009 151 369 378 10.7326/0003-4819-151-6-200909150-00001 19755362

[B61] Sommers LS Marton KI Barbaccia JC Randolph J Physician, nurse, and social worker collaboration in primary care for chronically ill seniors Arch Intern Med 2000 160 1825 1833 10.1001/archinte.160.12.1825 10871977

[B62] Van't Veer-Tazelaar P Smit F van Hout H van Oppen P van der Horst H Beekman A van Marwijk H Cost-effectiveness of a stepped care intervention to prevent depression and anxiety in late life: randomised trial Br J Psychiatry 2010 196 319 325 10.1192/bjp.bp.109.069617 20357310

